# Suicidal behaviors and interpersonal theory of suicide constructs among adolescent girls and emerging adult women with eating disorders: the moderating role of age

**DOI:** 10.3389/fpsyt.2025.1564384

**Published:** 2025-05-28

**Authors:** Amit Goldstein, Iris Shachar-Lavie, Orit Krispin, Roni Rom, Eitan Gur, Netta Horesh-Reinman, Yari Gvion

**Affiliations:** ^1^ Department of Psychology, Bar-Ilan University, Ramat Gan, Israel; ^2^ Eating Disorders Department, Schneider Children’s Medical Center, Petach Tikva, Israel; ^3^ Baruch Ivcher School of Psychology, Reichman University, Herzliya, Israel; ^4^ Eating Disorders Department, Sheba Medical Center, Tel Hashomer, Israel

**Keywords:** suicide, adolescents, eating disorders, anorexia, bulimia, suicidal behavior (SB)

## Abstract

**Background:**

Individuals with Eating Disorders (EDs) are at an elevated risk for suicidal behaviors (SB). The aim of this study was to explore the relationship between constructs of the Interpersonal Theory of Suicide (IPTS), SB and age among individuals with EDs.

**Methods:**

The study included 140 participants: 77 adolescent girls (ages 12-17) and 63 emerging adult women (ages 18-29). Participants completed a battery of psychological instruments assessing SB, ED symptom severity, non-suicidal self-injury (NSSI), perceived burdensomeness (PB), and thwarted belongingness (TB).

**Results:**

PB levels were higher among emerging adults compared to adolescents while TB levels were similar across groups. PB was significantly associated with SB among adolescents, but not among emerging adults. In contrast, TB was significantly associated with SB among emerging adults, but not among adolescents. No moderating effect of age was found in the relationship between NSSI and SB.

**Conclusion:**

These findings support the contribution of IPTS constructs (TB, PB) to SB among females with ED. Moreover, the identification of age-specific mechanisms by which IPTS constructs operate provides novel insight with potential clinical implications. Interventions for adolescents with EDs and SB may benefit from caregiver-focused strategies that reduce adolescents’ sense of burdensomeness, whereas interventions for emerging adults with EDs and SB may be improved by enhancing social integration and strengthening their sense of belongingness.

## 1 Introduction

Eating Disorders (EDs) are serious psychiatric conditions characterized by a preoccupation with body weight and maladaptive eating behaviors, and they are more prevalent among females (1). The typical onset of EDs occurs during adolescence (2, 3), and many individuals continue to experience significant ED symptomatology for years or even decades, sometimes persisting into late adulthood (4–7). A shorter duration of illness is associated with more favorable outcomes (8, 9) while a duration exceeding five years is linked to less favorable outcomes (10). Females with EDs are at particularly high risk for suicidal behaviors (SB) and death by suicide (11–13). Beyond ED-specific vulnerabilities, adolescents more broadly are recognized as being at increased risk for SB, driven by a constellation of clinical and psychosocial factors, such as sleep disturbances, emotional dysregulation, depressive symptoms, and exposure to adverse childhood experiences (14–16). These findings underscore the importance of early detection and integrated interventions that address emotional, behavioral, and environmental vulnerabilities.

Among individuals with EDs, both age and illness duration have been suggested as moderators of the relationship between ED symptom severity and suicidal behavior (17). The current study focuses on adolescents (ages 12–17) and emerging adults (ages 18–29)—a life stage that has increasingly been recognized as distinct from both adolescence and full adulthood. According to Arnett et al. (18) emerging adulthood is a developmental period marked by identity exploration and instability, with unique psychosocial challenges that may influence suicide risk. This broader age categorization (up to 29 years) is supported by recent literature that extends the boundaries of emerging adulthood in the context of mental health (18).

The Interpersonal-Psychological Theory of Suicide (IPTS) offers a well-established framework for understanding suicidal behavior, emphasizing the role of two interpersonal constructs—perceived burdensomeness (PB) and thwarted belongingness (TB)— as core contributors to suicidal desire (19–22). PB refers to the perception that one is a burden to others, while TB reflects feelings of isolation and a lack of meaningful social connections.

However, the desire to die is insufficient for suicide attempts; according to IPTS, individuals must also acquire the capability to enact lethal self-harm (21). This capability is theorized to develop through repeated exposure to painful and provocative experiences (PPE), which increase pain tolerance and reduce fear of death (22, 23). In individuals with eating disorders (EDs), the acquired capability for suicide may be especially pronounced due to engagement in behaviors such as vomiting, starvation, and compulsive exercise, which may function as PPEs (24–26). Another salient pathway is Non-Suicidal Self-Injury (NSSI), defined as the deliberate, self-inflicted harm to one’s body tissue without suicidal intent, which is notably prevalent in ED populations, particularly among females (approximately 72%) (27–31). Within the IPTS framework, NSSI is viewed as a salient PPE that fosters habituation to pain and facilitates progression toward lethal self-harm (20, 32). Research supports a strong association between NSSI and acquired capability for suicide (33, 34) Although much of this work has focused on adult and undergraduate samples, NSSI may be particularly influential during adolescence, when it is among the most common PPEs (35, 36). Given its theoretical and empirical relevance, NSSI was included in the present study alongside PB and TB to examine its contribution to suicidal behaviors among individuals with EDs. Dodd et al. (37) further support this inclusion, reporting a robust association between NSSI and acquired capability for suicide in women with EDs.

Although PB and TB are recognized as transdiagnostic constructs across psychiatric disorders, their manifestation may have unique features within ED populations. Individuals with EDs frequently experience interpersonal challenges, such as intense family involvement, caregiver burden, social withdrawal due to body image concerns, and prolonged illness-related isolation. These experiences may intensify feelings of burdensomeness and thwarted belongingness. Therefore, examining PB and TB among individuals with EDs is crucial for a nuanced understanding of suicide risk within this group and for informing targeted intervention strategies.

Previous research suggests strong support for PB and TB as reliable predictors of suicidal ideation among adults as well as adult individuals with EDs (32, 38). For instance, thwarted belongingness has been found to predict perceived burdensomeness particularly among females, thereby increasing suicidal ideation (39). Adult women with EDs often report loneliness (40), diminished pleasure in social interactions (41), and elevated isolation in chronic EDs (42). They also often describe themselves as unworthy, withdrawing from both family and social networks (43). A recent mini review suggests an association between PB, but not TB, and SB among adults with EDs (44). In line with that, it was found that caregivers of patients with EDs who purge report higher caregiver burden (45), which may further reinforce patients’ perceptions of being a burden.

While prior studies have highlighted the roles of PB, TB, and NSSI in suicide risk, the current study focuses specifically on age group as a moderator, examining whether the associations between these interpersonal constructs and suicidal behaviors differ between adolescents and emerging adults with EDs. Although other mediators such as ED severity or caregiver burden are acknowledged, they are not examined empirically in this study.

Most prior research applying the IPTS to ED populations has concentrated on adults, leaving a gap in understanding these associations in adolescents, despite adolescence being the primary onset period for EDs and a time when social relationships are particularly salient. Preliminary evidence links PB and TB to suicidal ideation among adolescents without EDs (46, 47), but few studies have explored these constructs in adolescents with EDs. Moreover, no prior research has directly compared suicidal behaviors between adolescents and emerging adults in ED populations. Addressing this gap may deepen our understanding of the developmental trajectories of EDs and suicide risk, ultimately guiding developmentally appropriate prevention and intervention efforts.

### 1.1 Aims

The current study aims to explore the relationship between constructs of the IPTS, suicidal behavior, and age among individuals with EDs. Specifically, we aim to determine whether emerging adults with EDs exhibit higher levels of SB, PB, and TB compared to adolescents with EDs. Furthermore, in light of existing evidence linking these IPTS components with SB in non-ED adolescent samples, a secondary objective is to investigate how age and IPTS constructs, including PB, TB, and non-suicidal self-injury (NSSI), relate to suicidal behavior in ED populations. As this study is exploratory in nature, no specific hypotheses are proposed. Instead, we seek to identify patterns that may inform future research and clinical interventions targeting suicide risk in individuals with EDs across different age groups.

## 2 Materials and methods

### 2.1 Participants

A total of 140 female participants with EDs, aged 12-29 (M= 19.17, SD=3.59) were divided into two groups: (1) sixty-three emerging adult women (18-29) admitted to an ED treatment facility in a general hospital in Israel between 2018-2019, and (2) seventy-seven adolescents treated in an ED treatment facility in a children’s hospital in Israel between 2019-2020 (see **Table 1** for details). The relatively modest sample size reflects the complexity of recruiting individuals from clinical settings, particularly those experiencing acute eating disorders. Ethical considerations and the need for clinical stability limited the eligible pool. Furthermore, engaging both adolescents and emerging adults across two separate healthcare institutions required extensive coordination and contributed to recruitment challenges. However, given the small sample size and the potential reduction in statistical power, we employed several cautious procedures and analyses to enhance the robustness of our results (see Data Analysis).

**Table 1:** Demographic and clinic characteristics of the age groups (N = 140).

Variable	Emerging Adults *(n = 63)*	Adolescents *(n = 77)*	*Statistical analysis*
Age (years)	M = 24.19	M = 15.23	t = 12.14***
	SD = 5.70	SD = 1.49	
Illness Duration	M = 4.75	M = 1.13	t = 5.42***
	SD = 5.23	SD = .96	
Diagnosis^1^			*χ* ^2^ = 29.07***
n (%)			
AN-R	18 (28.6)	51 (66.2)	
AN-BP	22 (34.9)	7 (9.1)	
BN	15 (23.8)	5 (6.5)	
OSFED	8 (12.7)	14 (18.2)	

Values are presented as n (%) or means ± SD.

1. In all types of diagnosis (except OSFED), the proportion of diagnoses significantly differs between age groups (*ps* ≤ .004) in Z test of equality for proportions using Bonferroni correction.

2. Anorexia Nervosa-Restricting type (AN-R), Anorexia Nervosa binge eating/purging type (AN-BP), Bulimia Nervosa (BN), Other Specified Feeding and Eating Disorders (OSFED).

*** p < .001.

### 2.2 Measures

#### 2.2.1 Interviews and self-rating questionnaires

Data on age and country of birth were collected for both groups, while data on marital status, number of children, educational level and employment were collected only for the adult group. Psychiatric diagnoses were assigned according to DSM, 5^th^ edition (48). Criteria for EDs were based on clinician interviews (medical physicians and clinical psychologists) conducted during intake sessions with the participants.

#### 2.2.2 INQ

The Interpersonal Needs Questionnaire (INQ) (49) measures PB and TB. It is a 15-item scale designed to measure beliefs about how connected one feels to others (i.e., belongingness), and the extent to which one may feel like a burden on others (i.e., PB). Eight items assess the construct of TB and seven assess PB, as defined earlier in this paper (20). Participants indicate the degree to which each item is true for them on a 7-point Likert-type scale. Scores are coded such that higher values reflect higher levels of thwarted belongingness and perceived burdensomeness. Cronbach’s alpha for perceived burdensomeness has ranged from 0.92 to 0.96, and for thwarted belongingness, from 0.79 to 0.86 (49). In our sample, the Cronbach’s alpha coefficient was 0.84 for perceived burdensomeness and 0.82 for thwarted belongingness.

#### 2.2.3 SBQR

The Suicide Behaviors Questionnaire (SBQ-R) developed by Osman and colleagues (50) is composed of four items, each tapping a different dimension of suicidality: lifetime suicide ideation and/or suicide attempts; the frequency of suicidal ideation over the past twelve months; the threat of suicide attempts; self-reported likelihood of SB in the future. Items are scored on a five to seven-point scale and are summed up to an aggregate score with a cutoff score of 7 (general population score). In this study, SB refers to responses across all four items. An analysis of suicidal ideation and attempts was performed separately in order to evaluate each measure. Reliability score ranged between 0.87-0.88 in adolescents and 0.76-0.87 in the adult population. In our sample, Cronbach’s alpha coefficient was found to be 0.81.

#### 2.2.4 EDE-Q

The Eating Disorder Examination Questionnaire (EDE-Q) (51) is used to index current eating disorder symptom severity. This questionnaire contains 28 items covering core ED symptoms and related variables and includes four subscales: Restraint, Eating Concern, Shape Concern, and Weight Concern. For the Hebrew translation, Shape and Weight Concern form a single factor. Twenty-two items are scored on a 7-point Likert scale ranging from 0 (no days/not at all) to 6 (every day/markedly). The total score was used the current study, and higher scores are indicative of greater severity. The other six items require an open numerical response and were excluded from scoring. Internal consistency for the four subscales has been found to range from 0.75 (restraint) to 0.93 (shape concern) (52). In this study, Cronbach’s α was 0.81.

#### 2.2.5 DSHI

A modified version of the Deliberate Self-Harm Inventory (DSHI) (53, 54) was used to assess engagement in self-injurious behaviors. It addresses six different self-injury items throughout the lifetime: cutting the body, self-burning, carving into the skin, preventing the healing of wounds, and banging the head against hard objects. An additional item assesses whether the injury was severe enough to require medical treatment. Items are rated on a 4-point Likert-type scale for rating the frequency of each behavior, ranging from 0 (“never”) to 3 (“five times or more”). The total score was calculated as an average of the six items. Cronbach’s alpha has previously been reported to range from 0.82-0.83 (54). In our sample, Cronbach’s alpha coefficient was 0.83.

### 2.3 Procedure

The study was conducted in accordance with the 1989 revised Helsinki Declaration and received Institutional Review Board (IRB) approval from the medical centers involved in the study. Prior to recruitment, candidates were evaluated by the medical staff to ensure clinical stability for participation. Exclusion criteria included a BMI lower than 12, lack of language proficiency or cognitive limitation that impaired understanding of the study objectives and the research questionnaires, or a psychotic state that prevented independent completion of the study materials. Participants were approached by clinical psychologists (M.A. level), who explained the aims of the study. Written informed consent was obtained from all participants. For participants under the age of 18, written parental consent was also obtained. All interviews and questionnaires were administered in a single session.

### 2.4 Data analysis

For the preliminary analysis, the distributions of the quantitative variables were examined using skewness, kurtosis, and normal QQ plots. Although the distributions were approximately normal, we conducted additional robust analyses that are less sensitive to assumption violations due to the small sample size. Missing data were minimal (<5% across all variables) and were handled using listwise deletion. No data imputation procedures were applied. Differences between age groups (adolescents and emerging adults) in background variables were examined using t-tests (for age and illness duration) and chi-square tests (for diagnosis).

The following analyses were conducted:

1. Differences between age groups in study variables were examined using ANOVAs (for questionnaire scores) and chi-square tests (for dichotomous variables: suicidal ideation [no, yes], suicide attempts [no, yes]). For robustness, group differences were also tested using the Mann–Whitney U test, which produced similar results.
2. Pearson correlations were calculated to examine associations between study variables.
3. A hierarchical linear regression model was conducted to predict the level of suicidal behavior (SB). In the first step, age group, diagnosis, and questionnaire scores were entered as predictor variables. In the second step, interaction terms between age group (adolescents *vs*. emerging adults) and all other predictors were included. Variance inflation factor (VIF) values were within acceptable limits, with a maximum VIF of 4.21 (55). To enhance robustness, we estimated the significance of coefficients using a non-parametric bootstrap procedure. Bootstrap methods are particularly appropriate for small samples, as they do not rely on distributional assumptions and allow for more reliable inference regarding model parameters under limited sample conditions. All statistically significant effects from the original regression remained significant in the bootstrap analysis.
4. To test the effects of significant interactions, we used Model 1 of the PROCESS macro (v4.0).

## 3 Results

### 3.1 Preliminary analysis

Normal QQ plots for the quantitative variables indicated approximately normal distributions, with a maximum skewness value of 1.45 and a maximum kurtosis value of 1.41.

**Table 1** presents differences between the age groups (emerging adults versus adolescents) in illness duration, and diagnosis. As shown in the table, a significant difference was found between the groups in both age and illness duration. In addition, a significant difference was found in diagnostic distribution. Among adolescents, most participants (66.2%) were diagnosed with AN-R, whereas among emerging adults, fewer than one-third (28.6%) received this diagnosis. About one-third were diagnosed with AN-BP (34.9%) and approximately one-quarter with BN (23.8%).

### 3.2 Differences between study groups

**Table 2** presents the differences between age groups across the study variables. Among emerging adults, a higher percentage of suicide attempts was reported compared to adolescents. No significant difference was found between the groups in the percentage of individuals reporting suicidal ideation. With regard to suicidal behavior (SB), calculated as the sum score of SBQR, the emerging adult group reported higher levels than the adolescent group. A significant difference was also observed in Perceived Burdensomeness (PB), with emerging adults reporting higher levels compared to adolescents. However, no significant difference was found between the groups in Thwarted Belongingness (TB). Additionally, significant group differences were observed in Non-Suicidal Self-Injury (NSSI) and eating disorder (ED) severity, with emerging adults exhibiting higher levels on both measures.

**Table 2:** Study variables by age groups (N = 140).

Variables	Emerging Adults *(n = 63)*	Adolescents *(n = 77)*	*Statistical analysis*
Dichotomous variables *n (%)*			
Suicidal Ideation	11 (17.5%)	10 (13.0%)	*χ* ^2^ (1) = .54
Suicide Attempts	25 (39.7%)	2 (2.6%)	*χ* ^2^ (1) = 30.61 *P< .001*
Continuous variables *M (SD)*			
Perceived Burdensomeness	M = 4.19	M = 3.33	*F* = 6.20 P < .05
(PB)	SD = 1.81	SD = 2.17	*η* ^2^ = .043
Thwarted Belongingness	M = 3.96	M = 3.78	*F* = .53
(TB)	SD = 1.28	SD = 1.53	*η* ^2^ = .004
NSSI	M = 1.68	M = 1.36	*F* = 8.17 P < .01
	SD = .81	SD = .52	*η* ^2^ = .056
ED severity	M = 4.22	M = 3.55	*F* = 5.48 P < .05
	SD = 1.45	SD = 1.87	*η* ^2^ = .038
Suicidal behavior	M = 7.70	M = 4.49	*F* = 14.47 P < .001
	SD = 5.51	SD = 4.46	*η* ^2^ = .095

Values are presented as n (%) or means ± SD.

### 3.3 Correlations between study variables

**Table 3** displays Pearson correlations between the study variables, calculated separately for each group. Diagnoses of AN-R, AN-BP, and BN are presented as dichotomous variables (0 = without the diagnosis, 1 = with the diagnosis). Correlations involving these variables were calculated using point-biserial correlation, a variant of Pearson correlation used to assess the relationship between a continuous variable and a dichotomous variable. A significant correlation between a diagnosis variable and another variable indicates a notable difference between individuals with the diagnosis and those without it in terms of the associated continuous variable.

**Table 3:** Pearson correlations between variables (N = 140).

	1	2	3	4	5	6	7	8	9
1. ANR	–	–	–	-.09	.03	-.11	.00	-.17	.05
2. ANBP	–	–	–	.19^*^	-.07	.11	-.13	-.02	-.11
3. BN	–	–	–	.02	.11	.12	.22^*^	.43^**^	.23^*^
4. Illness Duration	.00	.10	-.06	–	.03	.37^**^	.10	.19	.06
5. NSSI	-.33^**^	.14	.16	.21^*^	–	.32^**^	.46^**^	.40^**^	.61^**^
6. ED severity	-.19	.04	.27^*^	-.01	.29^*^	–	.40^**^	.50^**^	.30^**^
7. PB	-.15	.02	.10	.02	.48^**^	.24^*^	–	.60^**^	.73^**^
8. TB	-.25^*^	.05	.06	.08	.27^*^	.18	.43^**^	–	.48^**^
9. SB	-.55^**^	.17	.25^*^	.20	.60^**^	.31^**^	.51^**^	.57^**^	–

The correlations above the diagonal line (in the right-top) were calculated among adolescents, whereas the correlations below the diagonal line (in the left-bottom) were computed among emerging adults. Diagnoses of ANR, ANBP, and BN are presented as dichotomous variables (0 = without the diagnosis, 1 = with the diagnosis). The correlations involving these variables are measured using Point-Biserial Correlation.

* p < .05. ** p < .01.

Among emerging adults, a diagnosis of AN-R was negatively associated with NSSI, TB, and suicidal behavior, indicating lower levels of these variables among participants with the AN-R diagnosis. In contrast, among adolescents, a diagnosis of AN-BP was positively associated with illness duration, suggesting a longer duration of illness among those with this diagnosis. A diagnosis of BN was positively associated with ED severity and suicidal behavior in emerging adults, and with PB, TB, and suicidal behavior in adolescents. These findings suggest that emerging adults diagnosed with BN tend to exhibit higher levels of ED severity and suicidal behavior, whereas adolescents diagnosed with BN show elevated levels of PB, TB, and suicidal behavior. Furthermore, illness duration was positively associated with NSSI among emerging adults and with ED severity among adolescents. A Fisher r-to-z transformation revealed a significant difference between the age group in the strength of the association between illness duration and ED severity (Z = 2.29, p = .011). Finally, NSSI, ED severity, PB, TB, and suicidal behavior were all positively associated with each other, with the exception of a non-significant association between ED severity and TB in the emerging adult group.

### 3.4 Regression for predicting suicidal behavior

The assumptions of the regression model were met, with VIF smaller than 5, absolute standardized residuals less than 3, maximum Cook’s Distance of 0.13, and normality of the distributed residuals, as indicated by the normal predicted probability (P-P) plot. **Table 4** presents the regression model predicting suicidal behavior. The first step was statistically significant and accounted for 60.4% of the variance in suicidal behavior, as measured by the SBQR. Examination of the coefficients revealed that NSSI, PB and TB each made a significant unique contribution to the model: higher levels of NSSI, PB, and TB were associated with greater suicidal behavior. The second step was also significant and contributed 4.6% to the explained variance. Examination of the interaction terms revealed two significant interaction effects: (1) an interaction between PB and age groups, and (2) an interaction between TB and age group. To probe these interactions, simple slope analyses were conducted using PROCESS Macro v4.0.

**Table 4:** Hierarchical regression analysis to predict suicidal behavior.

*Predictor*	*B*	*SE*	*Beta*	*Partial* *Correlations*	*R^2^*	*ΔR^2^*	*ΔF*
**Step I**					.604	.604	24.99***
Age Groups	.41	.33	.08	.11			
AN-R	-.40	.42	-.08	-.08			
AN-BP	.27	.40	.05	.06			
BN	.47	.38	.09	.11			
NSSI	1.87	.34	.36***	.44			
ED severity	-.13	.33	-.03	-.04			
PB	1.78	.37	.34***	.38			
TB	.86	.36	.17*	.21			
**Step II**					.650	.046	4.16**
NSSI X Age Groups	-.48	.70	-.07	-.06			
ED severity X Age Groups	.59	.66	.06	.08			
PB X Age Groups	-1.67	.73	-.19*	-.20			
TB X Age Groups	2.53	.70	.29***	.31			

Age groups is a dichotomous variable (0 = adolescents, 1 = emerging adults). The values shown in the first step are the values calculated before introducing the interactions.

* p < .05. ** p < .01, *** p < .001.

**Figure 1:**
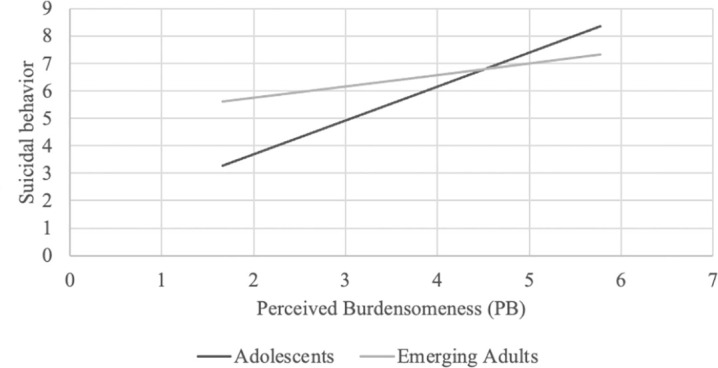
PB was significantly associated with suicidal behavior among the adolescent group, *B*=1.23, *SE*=.22, *p*<0.001, but not among the emerging adult group, *B*=.42, *SE*=.28, *p=*.130.

**Figure 2:**
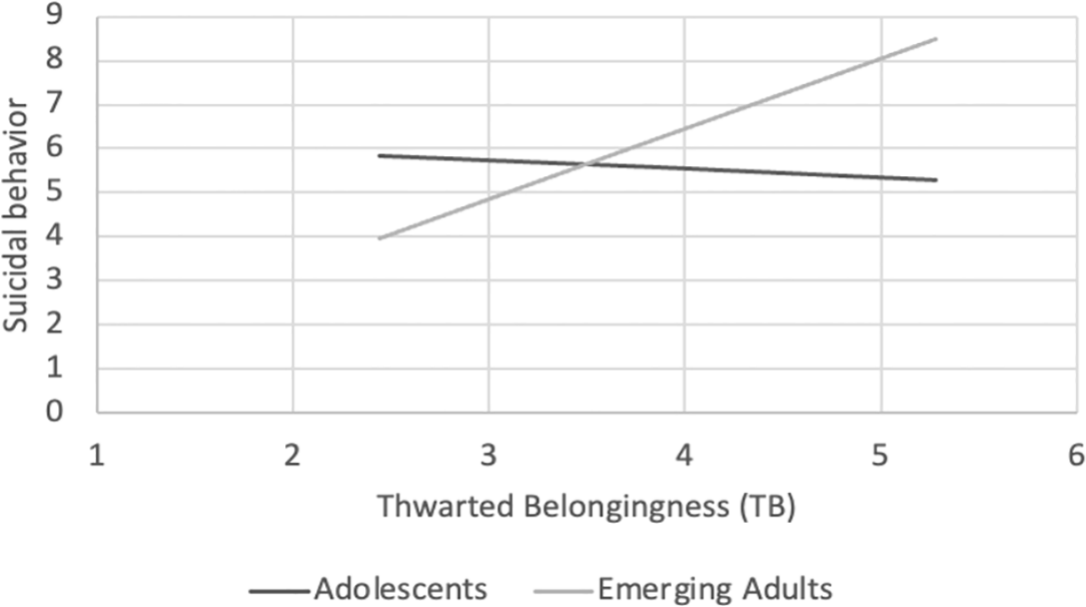
TB had a significant positive effect among emerging adults, *B*=1.60, *SE*=.36, *p*<0.001, but not among adolescents, *B*=-.18, *SE*=.34, *p*=.584.

Examining the interaction effects of age group with PB and with TB showed that PB was significantly associated with suicidal behavior among the adolescent group, *B* = 1.23, *SE* = .22, *p* <.001, but not among the emerging adult group, *B* = .42, *SE* = .28, *p* = .130 (see **Figure 1**). In contrast, TB was significantly positively associated with suicidal behavior among emerging adults, *B* = 1.60, *SE* = .36, *p* <.001, but not among adolescents, *B* = -.18, *SE* = .34, *p* = .584 (see **Figure 2**).

In addition, we considered the possibility that age might serve as a proxy for ED severity and illness duration. To explore this, we re-ran the same regression model, replacing ED severity with illness duration—both as a main effect in the first step and as an interaction term with age group in the second step. The results were similar to those of the initial regression: all effects that were significant in the original regression remained significant, and no additional significant effects emerged (First Step: *R^2^* = .607, *p* <.001; Coefficient: NSSI ( = .35, *p* <.001), PB ( = .34, *p* <.001), TB ( = .16, *p* = .023); Second Step: *R^2^* = .044, *p* = .004; Coefficient: PB X Age Group ( = -.18, *p* = .036), TB X Age Group ( = .30, *p* <.001).

## 4 Discussion

While the IPTS has been extensively studied in relation to suicide over the past fifteen years, research examining the association between its components and SB among individuals with EDs remains limited. Moreover, most existing studies have focused primarily on adult samples, with relatively little attention given to adolescent populations.

In the present study, emerging adults exhibited more severe ED symptoms and higher levels of SB compared to adolescents. This finding is consistent with previous research linking ED symptom severity to illness duration (10). Additionally, earlier studies have also indicated that SB tends to increase after the age of 18, particularly in women with chronic EDs who may experience hopelessness about treatment outcomes and despair about their condition. These emotional experiences can contribute to the development of depression, fear, and suicidal ideation (56, 57).

Both age groups displayed similar levels of thwarted belongingness (TB), but perceived burdensomeness (PB) was significantly higher among emerging adults. Individuals with EDs across age groups may encounter challenges in meeting age-relevant societal expectations, which can result in social exclusion. These challenges may manifest differently depending on developmental stage, such as adjusting to school, and peer interactions during adolescence, versus challenges related to employment, relationship, parenthood, and autonomy during adulthood (58). Although we discuss potential mechanisms such as illness duration, ED severity, and caregiver burden, these were not empirically tested in the current study and are presented as hypothetical.

The finding that the emerging adult group experienced higher levels of PB warrants further attention and investigation. Even after leaving the parental home, individuals with EDs often remain reliant on variant forms of caregiving—whether physical, emotional, or financial (59). Chronic caregiving for individuals with EDs can place a significant burden on caregivers, which may, in turn, reinforce the individual’s perception of being a burden (60, 61).

Adolescents with EDs may experience PB. However, given their developmental stage and complete dependence on caregivers, the emotional weight of feeling like a burden may be less acute. The current study examined whether age moderates the effects of PB, TB, and non-suicidal self-injury (NSSI) on SB. While NSSI demonstrated a strong association with SB, this relationship was not moderated by age, suggesting that the mechanisms linking NSSI to SB may differ from those underlying interpersonal constructs. NSSI is often conceptualized as an intrapersonal coping strategy involving emotional dysregulation, impulsivity, and habituation to pain (53, 62, 63), and may operate similarly across developmental stages. In contrast, PB and TB are embedded in social roles and interpersonal dynamics, which evolve significantly with age. This developmental shift may account for the age-specific associations observed with interpersonal risk factors, but not with NSSI.

### 4.1 PB

Our findings revealed an interesting pattern: the interaction between age group and PB showed that PB was significantly associated with SB among adolescents, but not among emerging adults. This suggests that PB may be a more salient predictor of SB during adolescence, potentially reflecting the interpersonal context of early- stage ED. During this developmental period, families are typically highly involved in caregiving, including monitoring meals and managing risky behaviors (64). While such involvement may foster a sense of belonging, it can also intensify feelings of being a burden.

Previous studies have highlighted the dominant presence of the ED in daily family life, often leaving limited emotional space for other family members, including siblings (60, 65, 66). Parents of adolescents with EDs frequently report heightened levels of anxiety, depression and social withdrawal (67–69). Adolescents may internalize these familial dynamics, perceiving themselves as the source of distress and disruption (29). This perception can lead to the belief that their death holds more value than their own life, as they may prioritize their family members’ needs over their own (70).

These findings underscore the importance of including families in treatment. Family-Based Therapy (FBT), which emphasizes improved communication, identification of triggers, and the development of healthier relationships with food, may help address these dynamics (71).

Interestingly, although PB levels were higher among emerging adults, PB was not associated with SB in this group. This may suggest that SB among emerging adults with EDs is more strongly influenced by chronic illness-related despair and social isolation than by perceptions of burdensomeness alone.

### 4.2 TB

Previous studies have indicated that PB is associated with SB in adult ED populations, while fewer studies have explored the role of TB (44). Although TB levels did not differ significantly between age groups in our study, TB was significantly associated with SB only among the emerging adults.

Adolescents often live with family members and are embedded in structured treatment settings (e.g., inpatient wards), where peer interactions with other youth experiencing similar ED-related challenges can foster a sense of community. Many also engage with online forums to connect with others facing similar struggles, which may provide social support, either toward recovery (72) or, conversely, by reinforcing disordered behaviors (73, 74). These connections may buffer the impact of TB on SB during adolescence.

In contrast, ED symptoms in emerging adults, are often chronic, involving severe physical and psychological impairments and recurrent hospitalizations (75). This might result in functional impairment across multiple life domains. Individuals with chronic ED frequently experience social difficulties, including isolation, loneliness and a significant reduction in social networks and romantic relationships (76, 77). It was found that 67% reported severe family problems, and 60% had to leave their jobs for a period of time, with their lives increasingly revolving around food and weight (59, 78). In severe cases, the ED itself may serve as a substitute for human connection, leading to emotional detachment and profound loneliness (78). This tragic situation can lead to depression, hopelessness and ultimately to SB (40, 41). These findings suggest that TB becomes a more prominent driver of SB during emerging adulthood. In such cases, it is essential to provide therapeutic interventions such as Interpersonal Therapy (IPT) that aim to improve their interpersonal functioning and enhance social support networks, thereby contributing to a reduction in ED symptoms (79).

Recent evidence suggests that emotional factors such as anger and hostility may also contribute to poorer clinical outcomes in individuals with eating disorders. Specifically, elevated levels of anger and hostility have been associated with increased rates of dropout from treatment and reduced engagement in follow-up evaluations (80). Similarly, impulsive behaviors of anger have been linked to less favorable treatment responses (81). Although we did not assess anger or hostility in the current study, these traits may contribute to SB through mechanisms involving emotional dysregulation and interpersonal dysfunction. Future research should explore these variables as potential moderators or mediators in the relationship between ED psychopathology and suicidality.

### 4.3 Limitations

Alongside our findings, several limitations should be acknowledged. First, difficulties in recruiting individuals with EDs resulted in a relatively small sample (n = 140). Although this is consistent with other clinical studies in the ED field, it may limit the generalizability of the results and reduce the statistical power to detect small or interaction effects. Second, the sample was composed exclusively of cisgender females. As a result, the findings may not extend to males or individuals with diverse gender identities. Given known gender differences in the presentation and interpersonal functioning of individuals with EDs, future research should aim to replicate and expand upon these findings in more gender-diverse populations. Doing so would enhance the inclusivity and applicability of clinical interventions targeting suicidal behaviors. Third, we did not examine potential mediators such as comorbidity with depression, which is known to be associated with negative interpersonal experiences and increased symptomatology. Finally, the cross-sectional design of the study precludes conclusions regarding causality.

## 5 Clinical implications

Our findings have important clinical implications for tailoring interventions according to developmental stage among individuals with eating disorders (EDs) at risk for suicidal behaviors (SB). The distinct patterns we identified suggest that perceived burdensomeness (PB) and thwarted belongingness (TB) contribute to SB through distinct mechanisms in adolescents versus emerging adults. Among adolescents with EDs, it is crucial to provide proper support to caregivers to help them manage the significant responsibilities associated with caring for a child with an ED. Family-Based Therapy (FBT) may particularly benefit from incorporating strategies that address and minimize adolescents’ feelings of burdensomeness (71). Practical adaptations might include emphasizing parental emotional availability, minimizing critical or blaming interactions, and reinforcing the adolescent’s valued role within the family system. Strengthening the perception of being a vital and supported family member may decrease feelings of burdensomeness and, in turn, reduce suicidal ideation and behaviors. In contrast, for emerging adults, our results highlight the greater clinical significance of thwarted belongingness. Thus, therapeutic approaches such as Interpersonal Therapy (IPT) should focus on enhancing social connectedness, rebuilding interpersonal networks, and addressing the profound loneliness and isolation often experienced by individuals with chronic EDs (79). Interventions might include working on repairing ruptured relationships, developing new social supports, and fostering a sense of belonging within community, academic, or occupational settings. Enhancing interpersonal functioning may serve as a protective factor against SB in this population. Overall, adapting intervention strategies to specifically target the interpersonal mechanisms most relevant to each developmental stage may optimize clinical outcomes and reduce the risk of suicidal behaviors in individuals with EDs.

## Data Availability

The raw data supporting the conclusions of this article will be made available by the authors, without undue reservation.
